# Evaluation of abnormal radiological findings in children aged 2 to 36 months followed by recurrent urinary tract infection: a retrospective study

**DOI:** 10.1080/0886022X.2016.1251460

**Published:** 2016-11-06

**Authors:** Cinar Ozen, Pelin Ertan, Feray Aras, Gul Gumuser, Mine Ozkol, Gonul Horasan Dinc

**Affiliations:** a Department of Pediatric Nephrology, Celal Bayar University, Manisa, Turkey;; b Department of Nuclear Medicine, Celal Bayar University, Manisa, Turkey;; c Department of Radiology, Celal Bayar University, Manisa, Turkey;; d Department of Public Health, Celal Bayar University, Manisa, Turkey

**Keywords:** Children, radiological findings, recurrent urinary tract infection, renal imaging

## Abstract

Our aim is to determine the rational usage of imaging techniques in order to prevent or minimize permanent renal damage in recurrent urinary tract infections (UTIs). This study was enrolled children aged between 2 and 36 months, following-up with the diagnosis of recurrent UTI. All children had ultrasonography (USG) and dimercaptosuccinic acid scanning, 39 of them had underwent on voiding cystourethrography. There were 133 children (87 girls, 46 boys) with the mean age of 32.82 ± 38.10 months included into the study. Forty-three kidney units were normal in ultrasonogram of which seven units had reflux whereas among 35 units with hydronephrosis 22 units had reflux. Sensitivity and specificity presence of hydronephrosis in ultrasonogram for prediction of reflux was 75.9% and 73.5%, respectively. There were 19 dilated ureters in ultrasonogram, and among them 14 had reflux. Sensitivity and specificity of presence with ureteral dilatation in ultrasonogram for prediction of reflux was found as 48.3% and 89.8%, respectively. The sensitivity of parenchymal thinning seen in ultrasonogram for the evaluation of renal parenchyma was 15.9%, whereas specificity was 98.2% .Sensitivity and specificity of dimercaptosuccinic acid for prediction of reflux was 51.6% and 72.3%, respectively. The normal ultrasonogram findings cannot rule out neither possibility of reflux presence nor development of renal scarring. Therefore, DMSA scanning has major role both in determination of parenchymal damage and prevention of scarring. Also we get an important result as ureteral dilatation seen in USG, related to presence of reflux.

## Introduction

Urinary tract infection (UTI) is one of the major clinical problems in childhood. Upper UTI (pyelonephritis) can lead to renal damage, hypertension, and end stage renal disease in future.^1^ It is more difficult to differentiate cystitis or pyelonephritis clinically, particularly in cases under two years of age. Additional laboratory and imaging methods are beneficial for diagnosis. Recurrent UTI (R.UTI) is defined as; two or more pyelonephritis, once pyelonephritis with one or more cystitis (lower UTI) or three or more cystitis.^2–4^ Vesicoureteral reflux (VUR), is an abnormal urine flow from bladder to kidneys. The frequency of VUR is approximately 1% in the general population whereas it raises up to 30–50% in children with recurrent UTI.^5^ Therefore, presence of reflux must be investigated in especially recurrent UTIs.^6^ A number of guides have been published to demonstrate the risk factors that can lead to renal damage in UTIs. The early diagnosis and appropriate treatment of the disease is important for the renal damage risk in future. Nowadays there are various imaging modalities for diagnostic evaluation but it involves different approaches. Although diagnostic imaging method is the proposed approach in the first febrile UTI, there are debates about which is the most appropriate approach.^3^ ^,^ ^4^ ^,^ ^7^ ^,^ ^8^ The purpose of this study is to determine the rational use of imaging techniques in order to prevent or to minimize permanent renal damage in recurrent UTIs.

## Materials and methods

After the local ethics committee approval, patients followed with the diagnosis of recurrent UTI in the Celal Bayar University Pediatric Nephrology Clinic between January 2012 and July 2015, aged 2–36 months were included into the study. Patient data were obtained retrospectively from charts in the nephrology clinic. The age, gender, demographics, physical examination findings, laboratory results, imaging studies, and treatment modalities were recorded. Also, status of circumcision, labial synechia presence, first febrile UTI age, total UTI number with fever or none, culture taking method, presence of hospitalization, duration of prophylaxis were noted. For the samples taken with catheter, colony number of 5 × 10^4^ with only one type of microorganism was considered significant.^4^ Parenchymal thickness, dilatation on upper urinary systems and ureters were noted in ultrasonography (USG). The patients with suspected VUR findings, were evaluated with voiding cystoureterography (VCUG). The degree of reflux was determined according to the international classification.^9^ ^,^ ^10^ Patients with missing data were excluded from the study. Technetium-99m-Dimercaptosucsinic asit (DMSA) imaging was performed using a standard protocol. The acute phase DMSA findings were evaluated according to number of hypoactive kidney areas separately for each renal unit thus each functional ratio was recorded. Four standard projections as anterior–posterior and posterior oblique images were obtained four hours after İ.V injection of Tc^99m^ DMSA (50 μCi/kg).^11^

Statistical Package for Social Sciences (SPSS) for Windows 15.0 (SPSS Inc., Chicago, IL) was used for statistical analysis. The data were presented by numbers, percentages, mean ± standard deviations. Kolmogorov Smirnov test has been used for checking normality assumption. Student’s *t*-test and Mann–Whitney *U* test were used for analysis of numerical data and chi-square test was for nominal data. Sensitivity, specificity, positive predictive value, and negative predictive value were also calculated. Kappa values and hypothesis testing were applied for proven of diagnostic tests. Type 1 error as 5% has been accepted.

## Results

The study was held with 133 cases (totally 266 renal units). The mean age of the patients was 32.82 ± 38.10 months, 87 of them were female and 46 were male. Median age at first febrile UTI was 16.69 ± 12.97 (0–36) months for girls and 4.70 ± 6.76 (0–25) months for boys. The number of febrile UTI episodes was higher in the first year for boys (Table 1). All cases had urinary USG and DMSA scanning. VCUG was performed for 39 cases (78 renal units).

**Table 1.** Descriptive characteristics of study population.

	Boys (*n* = 46)	Girls (*n* = 87)	Total (*n* = 133)
Age, months, mean ± SD (min–max)	12.38 ± 22.72 (2–36)	44.87 ± 40.24 (2–36)	32.82 ± 38.10
Labial synechia (%)	–	2 (%2.3)	–
Circumcision (%)	6 (%13.3)	–	–
First febrile UTI age, months, mean ± SD (min–max)	4.70 ± 6.76 (0–25)	16.69 ± 12.97 (0–36)	12.54 ± 12.57 (0–36)
Total number of febrile UTI, mean ± SD (min–max)	1.13 ± 1.10 (0–5)	1.56 ± 1.10 (0–6)	1.41 ± 1.12 (0–6)
Total number of a febrile UTI, mean ± SD (min–max)	2.17 ± 1.35 (0–6)	1.64 ± 1.30 (0–6)	1.83 ± 1.34 (0–6)
Prophylaxis duration, months, mean ± SD (min–max)	8.57 ± 6.53 (0–26)	3.14 ± 4.79 (0–20)	5.02 ± 6.02 (0–26)

Forty-three kidney units were normal in USG of which 7 units had VUR whereas in 35 units with abnormal USG (hydronephrosis) 22 units had VUR. In other words, among total 29 renal units with VUR, 22 of them had hydronephrosis in USG. Sensitivity and specificity of presence of hydronephrosis in USG for prediction of VUR was 75.9% and 73.5%, respectively (Table 2, kappa = 0.473, *p* = .001).

**Table 2.** The accuracy and reliability of diagnostic test.

Diagnostic test	Gold standard test	+/+^a^ n (%)	+/−^a^ n (%)	−/+^a^ n (%)	−/−^a^ n (%)	Total (n) (%)	Sensitivity (%)	Specificity (%)	+ PV	−PV	Kappa value	*p*
USG PT	DMSA lesion (+)	7 (2.6)	4 (1.5)	37 (13.9)	218 (82.0)	266	15.9	98.2	63.6	85.5	0.202	**.001**
USG HN (+)	VCUG VUR (+)	22 (28.2)	13 (16.7)	7 (9.0)	36 (46.2)	78	75.9	73.5	62.9	83.7	**0.473**	**.001**
VCUG VUR (+)	DMSA lesion (+)	16 (20.5)	13 (16.7)	15 (19.2)	34 (43.6)	78	51.6	72.3	55.2	69.4	0.242	**.032**
USG UD (+)	VCUG VUR (+)	14 (17.9)	5 (6.4)	15 (19.2)	44 (56.4)	78	48.3	89.8	73.7	74.6	**0.410**	**.001**

a Diagnostic test diagnosis (positive or negative)/Gold standard test diagnosis.

HN: hyronephrosis; PT: parenchymal thinning; UD: ureteral dilatation; +PV: positive predictive value; −PV: negative predictive value.

There were 19 dilated ureters in USG, among them 14 had VUR. Sensitivity and specificity of presence of ureteral dilatation in USG for prediction of VUR was found as 48.3% and 89.8%; respectively (Table 2, kappa = 0.410, *p* = .001).

There were parenchymal thinning seen in ultrasound exam of 11 renal units, among them seven had parenchymal lesion on DMSA. The sensitivity of parenchymal thinning seen in USG for the evaluation of parenchymal lesion on DMSA was 15.9%, whereas specificity was 98.2% (Table 2, kappa = 0.202, *p* = .001).

There were 31 renal units with parenchymal lesion on DMSA and among them 16 had reflux. On the other hand, 13 renal unit had also reflux although they were involved into the group without abnormal DMSA (*n* = 47). Sensitivity and specificity of DMSA for prediction of VUR was 51.6% and 72.3%, respectively (Table 2, kappa =0.242, *p* = .032).

## Discussion

American Academy of Pediatrics (AAP) has been updated the diagnostic and management approach for the first febrile UTI between 2 and 24 months children in 2011. USG may lead over- under diagnosis at an early stages in this age group who had first febrile UTI, so they have reported that, it would be better to do after infection. Also, they did not recommend to do routine VCUG or DMSA in children with normal USG, who had a first febrile UTI.^4^ According to data from National Institute for health and Clinical Excellence (NICE), it is not recommended to do DMSA and/or VCUG in children under six months who had febrile UTI unless any USG findings, atypical or R.UTI. Also, it is not recommended to do USG exam in acute phase, in children after six months unless having atypical or R.UTI.^2^ ^,^ ^3^

Trisha et al.^12^ reported that the presence of reflux in 25% of patients having normal ultrasound, undergoing the first febrile UTI. It was emphasized that only USG exam would not be sufficient for determining diagnosis and follow-up due to the fact that, there may be severe degrees of reflux or need for surgery, even if the normal USG in children with recurrent pyelonephritis.

In a retrospective study evaluating diagnostic value of different imaging modalities, it has been pointed out that the normal ultrasound findings could be able to rule out high-grade reflux in children with a first febrile UTI.^13^ Leroy et al. evaluated 118 children with recurrent UTI, they searched for the specify of USG in the determination of VUR. The ratio of VUR had been detected as 27%, of which 7% had grade 3–5 VUR.

There had been shown to be a significant relationship (correlation) between reflux and ureteral dilatation, especially with high-grade VUR. Therefore, they reported the sensitivity and specificity of ureteral dilatation in USG for determining of high-grade VUR as 73% and 88%; respectively^14^.

In the present study 75.9% sensitivity and 73.5% specificity of presence of hydronephrosis have been found by ultrasound for determination of reflux, thus concluded that the normal USG findings did not exclude the diagnosis of reflux or pyelonephritis similar to the literature. However, the sensitivity of ureteral dilatation in ultrasound was found as 48.3% while specificity was 89.8% for detecting reflux (Table 2). Relatively higher agreement has been found between VUR diagnosis and ureteral dilatation (kappa = 0.410, *p* = .001). Also same agreement was shown between VUR diagnosis and hydronephrosis (kappa = 0473, *p* = .001). Therefore in the presence of ureteral dilatation or hydronephrosis in children with recurrent UTI, it was thought to investigate reflux at first would be more appropriate.

In the study, conducted by Nickavar et al. any significant correlation between ultrasound findings and DMSA was shown in acute pyelonephritis. Ultrasound was assessed as inadequate in the diagnosis of acute pyelonephritis (34% sensitive, 53% specific).^13^ Of the patients with lesions on DMSA, 30–50% may have signs of acute pyelonephritis in USG,^13^ ^,^ ^15^ ^,^ ^16^ therefore it was reported that normal ultrasound findings could not exclude acute pyelonephritis.^13^ ^,^ ^17^ Lavocart et al. investigated the importance of DMSA screening in patients with pyelonephritis, and showed the presence of USG abnormalities in 45% and parenchymal changes of DMSA in 93% of the children with febrile UTI. According to this; normal USG could not exclude the renal parenchymal lesion.^18^ ^,^ ^19^

In the present study, the sensitivity of renal parenchymal thinning in the ultrasound evaluation was found as 15.9%, specificity was determined as 98.2%. Although there seems to be statistically significant relation between USG and DMSA findings for the renal parenchymal evaluation, it has found low consistency due to its low kappa value (kappa = 0.202, *p* = .001). Therefore, we could not show significant correlation between the ultrasound findings with DMSA in our study (Table 2). These results indicated that it will not be enough just to trust ultrasonographic findings for the evaluation of renal parenchyma in patients with UTI, DMSA must be performed in the case of clinical suspicion.

It has been reported that, there was reflux in 27–70% of patients with abnormal DMSA.^13^ ^,^ ^17^ ^,^ ^20^ In his study of Nickavar et al. reflux was detected in 35.7% patients with DMSA lesions (sensitivity and specificity is low).^13^ In the study done by Bhatnagar with 89 children for the evaluation of relationship between UTI, VUR, and renal scarring; they found that 15 children among 23, had renal scarring although they did not have VUR.^7^ In our study there was 50% of reflux seen among the renal units having parenchymal lesion on DMSA. DMSA sensitivity and specificity for the evaluation of VUR was detected in 51.6% and 72.3%; respectively (Table 2). Although it seems to be statistically significant relation between VCUG and DMSA findings for the reflux determination, it has found low consistency due to its low kappa value (kappa = 0.242, *p* = .032).

Our study was discussed at DMSA, voiding cystourethrography and ultrasound triangle. Those imaging methods are useful and easily accessible. However, one of the new imaging methods is magnetic resonance urography (MRVCUG). MRVCUG is a reliable and safe diagnostic tool to determine renal scars in VUR patients. It can substitute for Tc-DMSA scintigraphy, particularly in patients requiring follow-up scanning and, consequently, considerable radiation exposure. MRVCUG is a noninvasive and non-radiating imaging method and it is alternative to standard VCUG for diagnosing and managing patients with VUR.^21^ Therefore, this method may be preferred for the future.

## Conclusions

Early diagnosis is important for renal damage in the following period. The normal findings of USG cannot exclude reflux or risk of renal scarring. Therefore DMSA scanning has major role both in determination of parenchymal damage and prevention of scarring. Also we get an important result as ureteral dilatation seen in USG, related to presence of reflux. Thus, it was thought that it is necessary to investigate the presence of vesicouretheral reflux if hydronephrosis or ureteral dilatation was present in USG.
